# Coagulation factor V is a T-cell inhibitor expressed by leukocytes in COVID-19

**DOI:** 10.1016/j.isci.2022.103971

**Published:** 2022-02-22

**Authors:** Jun Wang, Prasanti Kotagiri, Paul A. Lyons, Rafia S. Al-Lamki, Federica Mescia, Laura Bergamaschi, Lorinda Turner, Michael D. Morgan, Fernando J. Calero-Nieto, Karsten Bach, Nicole Mende, Nicola K. Wilson, Emily R. Watts, Cambridge Institute of Therapeutic Immunology and Infectious Disease-National Institute of Health Research (CITIID-NIHR) Covid BioResource Collaboration, Patrick H. Maxwell, Patrick F. Chinnery, Nathalie Kingston, Sofia Papadia, Kathleen E. Stirrups, Neil Walker, Ravindra K. Gupta, David K. Menon, Kieren Allinson, Sarah J. Aitken, Mark Toshner, Michael P. Weekes, James A. Nathan, Sarah R. Walmsley, Willem H. Ouwehand, Mary Kasanicki, Berthold Göttgens, John C. Marioni, Kenneth G.C. Smith, Jordan S. Pober, John R. Bradley

**Affiliations:** 1 Department of Medicine, University of Cambridge, Addenbrookes Hospital, Box 157, Hills Rd, Cambridge CB2 0QQ, UK; 2 Cambridge Institute of Therapeutic Immunology and Infectious Disease, Jeffrey Cheah Biomedical Centre, University of Cambridge, Cambridge CB2 0AW, UK; 3 Cambridge University Hospitals NHS Foundation Trust, Addenbrooke’s Hospital, Cambridge CB2 0QQ, UK; 4 Wellcome Sanger Institute, Wellcome Genome Campus, Hinxton, Cambridge, UK; 5 Cancer Research UK –Cambridge Institute, Robinson Way, Cambridge CB2 0RE, UK; 6 Department of Haematology, Wellcome and MRC Cambridge Stem Cell Institute, University of Cambridge, Cambridge, Cambridgeshire CB2 0AW, UK; 7 Department of Pharmacology, University of Cambridge, Cambridge CB2 1PD, UK; 8 Centre for Inflammation Research, Queen’s Medical Research Institute, University of Edinburgh, Edinburgh EH16 4TJ, UK; 9 NIHR BioResource, Cambridge University Hospitals NHS Foundation, Cambridge Biomedical Campus, Cambridge CB2 0QQ, UK; 10 Department of Clinical Neurosciences, School of Clinical Medicine, University of Cambridge, Cambridge Biomedical Campus, Cambridge CB2 0QQ, UK; 11 Medical Research Council Mitochondrial Biology Unit, University of Cambridge, Cambridge Biomedical Campus, Cambridge CB2 0XY, UK; 12 Department of Haematology, School of Clinical Medicine, University of Cambridge, Cambridge Biomedical Campus, Cambridge CB2 0QQ, UK; 13 Department of Public Health and Primary Care, School of Clinical Medicine, University of Cambridge, Cambridge Biomedical Campus, Cambridge CB2 0QQ, UK; 14 Department of Histopathology, Cambridge University Hospitals NHS Foundation Trust, Cambridge CB2 0QQ, UK; 15 Medical Research Council Toxicology Unit, University of Cambridge, Gleeson Building, Tennis Court Road, Cambridge CB2 1QR, UK; 16 Department of Pathology, University of Cambridge, Tennis Court Road, Cambridge CB2 1QP, UK; 17 Royal Papworth Hospital NHS Foundation Trust, Papworth Road, Cambridge CB2 0AY, UK; 18 NHS Blood and Transplant, Cambridge Biomedical Campus, Cambridge CB2 0PT, UK; 19 EMBL-EBI, Wellcome Genome Campus, Hinxton, CB10 1SD, UK; 20 Department of Immunobiology, Yale University School of Medicine, New Haven, CT 06519, USA

**Keywords:** Immunology, Microbiology, Omics, Transcriptomics

## Abstract

Clotting Factor V (FV) is primarily synthesized in the liver and when cleaved by thrombin forms pro-coagulant Factor Va (FVa). Using whole blood RNAseq and scRNAseq of peripheral blood mononuclear cells, we find that FV mRNA is expressed in leukocytes, and identify neutrophils, monocytes, and T regulatory cells as sources of increased FV in hospitalized patients with COVID-19. Proteomic analysis confirms increased FV in circulating neutrophils in severe COVID-19, and immunofluorescence microscopy identifies FV in lung-infiltrating leukocytes in COVID-19 lung disease. Increased leukocyte FV expression in severe disease correlates with T-cell lymphopenia. Both plasma-derived and a cleavage resistant recombinant FV, but not thrombin cleaved FVa, suppress T-cell proliferation *in vitro*. Anticoagulants that reduce FV conversion to FVa, including heparin, may have the unintended consequence of suppressing the adaptive immune system.

## Highlights

• Neutrophils, T-cells including T regulatory cells, and monocytes produce Factor V
• Leukocyte expression of Factor V increases in COVID-19
• Factor V suppresses T conventional cell proliferation *in vitro*
• Leukocyte Factor V expression correlated with lymphopenia in COVID-19

## Graphical abstract

**Figure:**
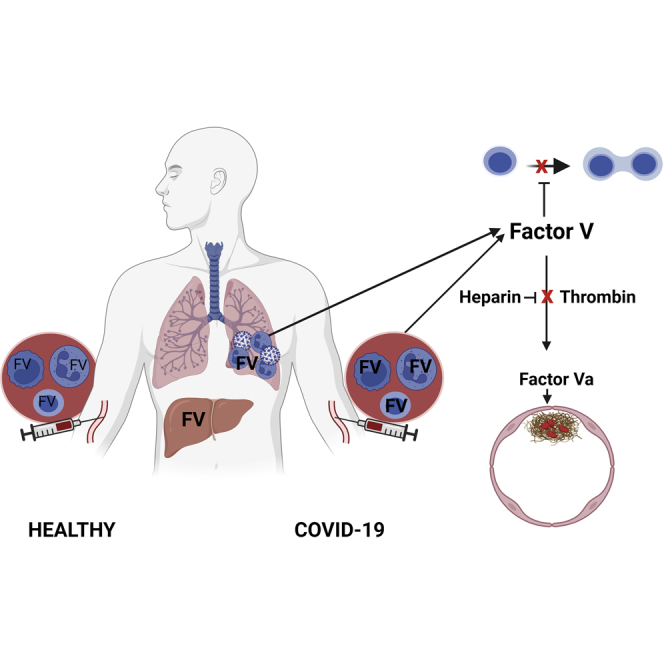

## Introduction

Dysregulation of both the immune (Zhou et al., 2020) and coagulation systems (Bikdeli et al., 2020) occurs in severe COVID-19 infection. Immunological responses include T-cell lymphopenia, which we have found can persist for months after the initial illness (Bergamaschi et al., 2021). Coagulopathy is an important cause of morbidity and mortality in patients with COVID-19, and a marked increase in circulating Factor V (FV) activity has been reported in patients with severe COVID-19, associated with increased risk of thromboembolism (Stefely et al., 2020).

FV is primarily produced by the liver, and circulates as a 330 kD inactive anticoagulant form composed of a heavy chain, B domain, and light chain. The B domain contains basic (aa 963 to 1008) and acidic (aa 1492 to 1538) regions, which are predicted to bind together, exposing the intervening motif on the surface of the folded structure. The B domain is required for the anticoagulant activity of FV(Cramer and Gale, 2012; Thorelli et al., 1998). Thrombin, and in the presence of phospholipids, Factor Xa(Monkovic and Tracy, 1990) sequentially cleave FV at Arg^709^, Arg^1018^ and Arg^1545^, releasing a cleaved B domain to create a heavy and light chain dimer, designated as FVa, that can bind factor Xa and act as a pro-coagulant.

Production of FV by T-cells (Shen et al., 1993) and monocytes(Dashty et al., 2012) has been previously reported. We were interested to learn whether leukocyte derived FV may increase in COVID-19, and if so what the functional consequences of this may be. We report here that circulating and lung-infiltrating leukocytes are a source of increased FV in patients with COVID-19. We further show that intact FV, but not FVa, inhibits T-cell proliferation *in vitro*. We propose that neutrophil-, monocyte- and Treg-derived Factor V may be an important cause of the dysregulated T-cell response to SARS-CoV-2.

## Results

### FV is produced by circulating blood cells and this is increased in hospitalized patients with COVID-19

**Figure 1:**
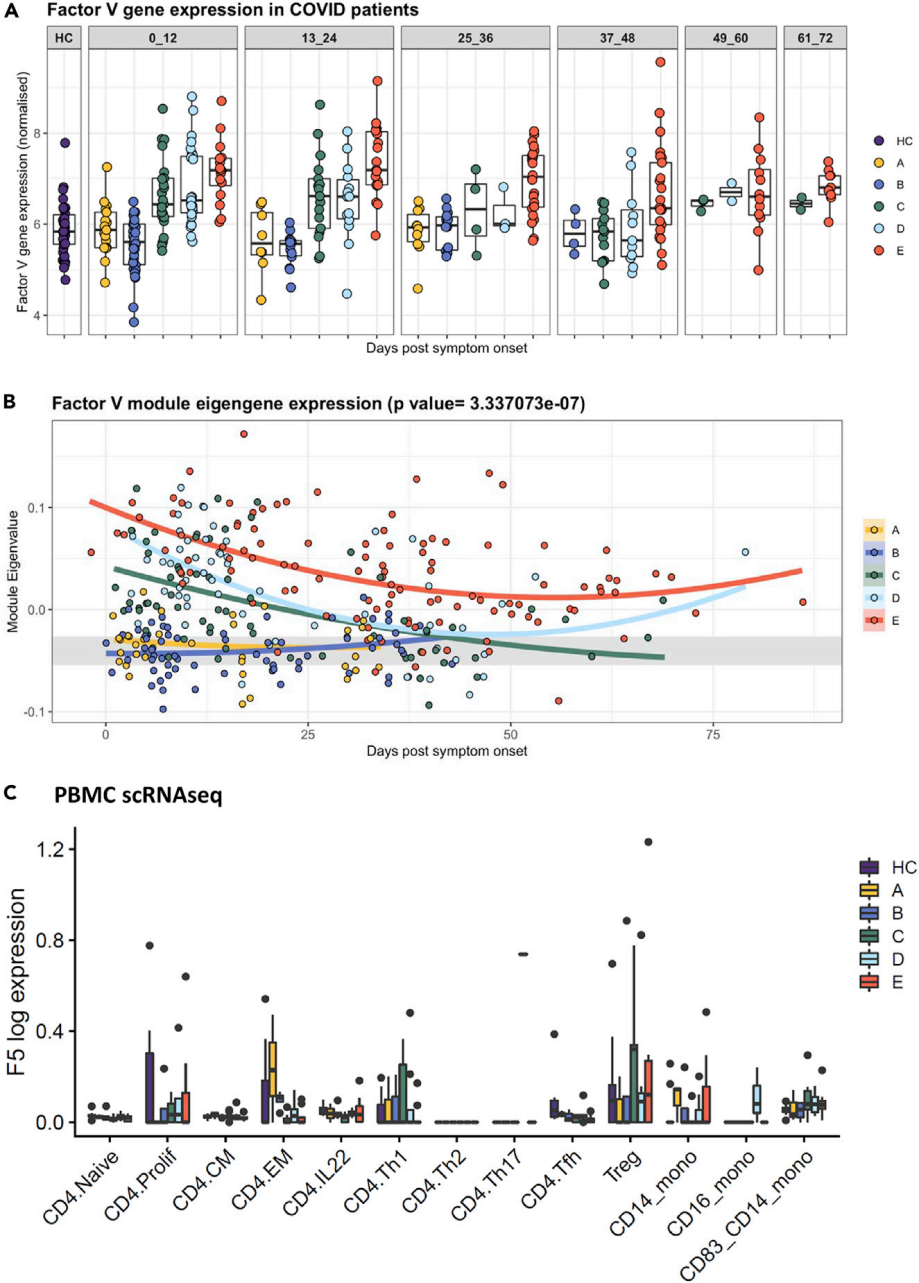
Increased FV gene expression in circulating leukocytes in hospitalized patients with COVID-19 (A) Peripheral blood cells from healthy controls, healthcare workers and patients with COVID-19 express FV. Individuals represented by dots are grouped into 12 days time periods after onset of symptoms or a positive swab in asymptomatic healthcare workers (HCW). HC, healthy controls; A, HCW screening asymptomatic; B, HCW screening symptomatic; C, hospitalized mild disease; D, hospitalized requiring oxygen; E, hospitalized, intensive care. 0 to 12 days C versus HC p = 0.003; D versus HC p = 0. 0000008; E versus HC p < 0.00001. 13 to 24 days C versus HC p = 0.0054; D versus HC p = 0.045; E versus HC p < 0.00001. 25–36 days E versus HC p = 0.00003. 49 to 60 days E versus HC p < 0.0000. 61 to 72 days E versus HC p = 0.0006. Box plot indicates interquartial range. (B) Weighted gene co-expression network analysis identified a module containing group of genes co-expressed with FV, in which FV is a hub gene and its expression correlates strongly with genes expressed in neutrophils (Figure S1). Mixed-effects model with quadratic time trend showing the longitudinal expression of the FV module over time, grouped by severity. Gray band indicates the IQR of HCs. A significant effect of time versus severity group interaction term (p = 3.33e-07) indicates that disease severity has a significant effect on longitudinal expression. (C) scRNAseq of PBMCs derived from HC, HCW and patients with COVID-19 showed the highest expression of FV in CD4^+^, FoxP3+ Tregs, with expression also detected in monocytes and at lower levels in other CD4 cell subsets. FV expression was increased in Tregs versus CD4^+^ Naive cells in healthy controls (p = 8.33 × 10^−4^), and in severe COVID-19 versus healthy controls in CD14 monocytes (p = 0.016) but not other cell subsets. See also Figures S1, S3 and Table S1. Boxes denote IQR with median shown as horizontal bars. Whiskers extend to 1.5x the IQR; outliers are shown as individual points.

Analysis of the transcriptome of peripheral blood cells from 246 healthy controls and patients with COVID-19 shows expression of FV, and expression is increased in patients with more severe disease for at least 72 days after onset of symptoms (Figure 1A). Weighted gene co-expression network analysis was performed on the transcriptomes to create distinct modules comprising non-overlapping co-expressed genes. The module containing FV also contained genes strongly expressed in neutrophils (Figure S1). In addition, FV was a “hub gene” meaning its expression closely mirrored that of the module eigengene, which is a single expression profile summarizing all genes within the module. The genes in the ‘FV module’ were also analyzed using the Enrichr enrichment analysis tool and this has identified enrichment for complement (adjusted p value = 0.00001339, OR 4.63), angiogenesis (adjusted p value = 0.002152, OR 8.14), and coagulation (adjusted p value = 0.0002792, OR 4.60). GO enrichment analysis has confirmed enrichment for genes involved in neutrophil activation and immune and inflammatory responses. This data is included as Table S1.

Correlation of the FV module eigengene expression with severity of COVID-19 is highly significant (Figure 1B). FV module expression is similar to healthy controls in asymptomatic or mildly symptomatic individuals in the community. In hospitalized patients with mild disease FV module expression is elevated at presentation, but declines as patients recover (Figure 1B). In patients with severe disease FV module expression is elevated at presentation and increased levels persists for several weeks. Analysis of the absolute number of the cell types that were found to express FV over time shows that CD4 cells and Tregs are suppressed early in disease, whereas neutrophil counts are elevated and remain high in patients with severe disease (Figure S2).

Analysis of peripheral blood cells for expression of other coagulation factors showed expression of Factor XIIIa and low levels of Factor XII but not other coagulation factors (data not shown).

scRNAseq was performed on peripheral blood mononuclear cells (PBMCs) derived from 47 individuals recruited in Cambridge UK for whom FV data is available. These volunteers form part of a larger cohort of 130 volunteers in whom scRNAseq was performed (Stephenson et al., 2021). Tregs and monocytes express FV in patients with COVID-19. Tregs (CD4^+^, FoxP3+) had the highest expression of FV, with expression also detected in monocytes and lower levels in other CD4 cell subsets (Figure 1C).

Analysis of the Blueprint (https://www.blueprint-epigenome.eu/) database of hematopoietic genome-scale datasets from healthy volunteers shows the highest level of FV expression in neutrophils, eosinophils, Tregs and monocytes (Figure S3).

### FV mRNA expression correlates with protein expression and parameters of disease severity and lymphopenia

To determine whether increased peripheral blood cell FV mRNA levels is associated with FV protein expression we assayed plasma FV levels and performed liquid chromatography – mass spectrometry on neutrophil lysates from healthy controls and patients with severe COVID-19.

**Figure 2:**
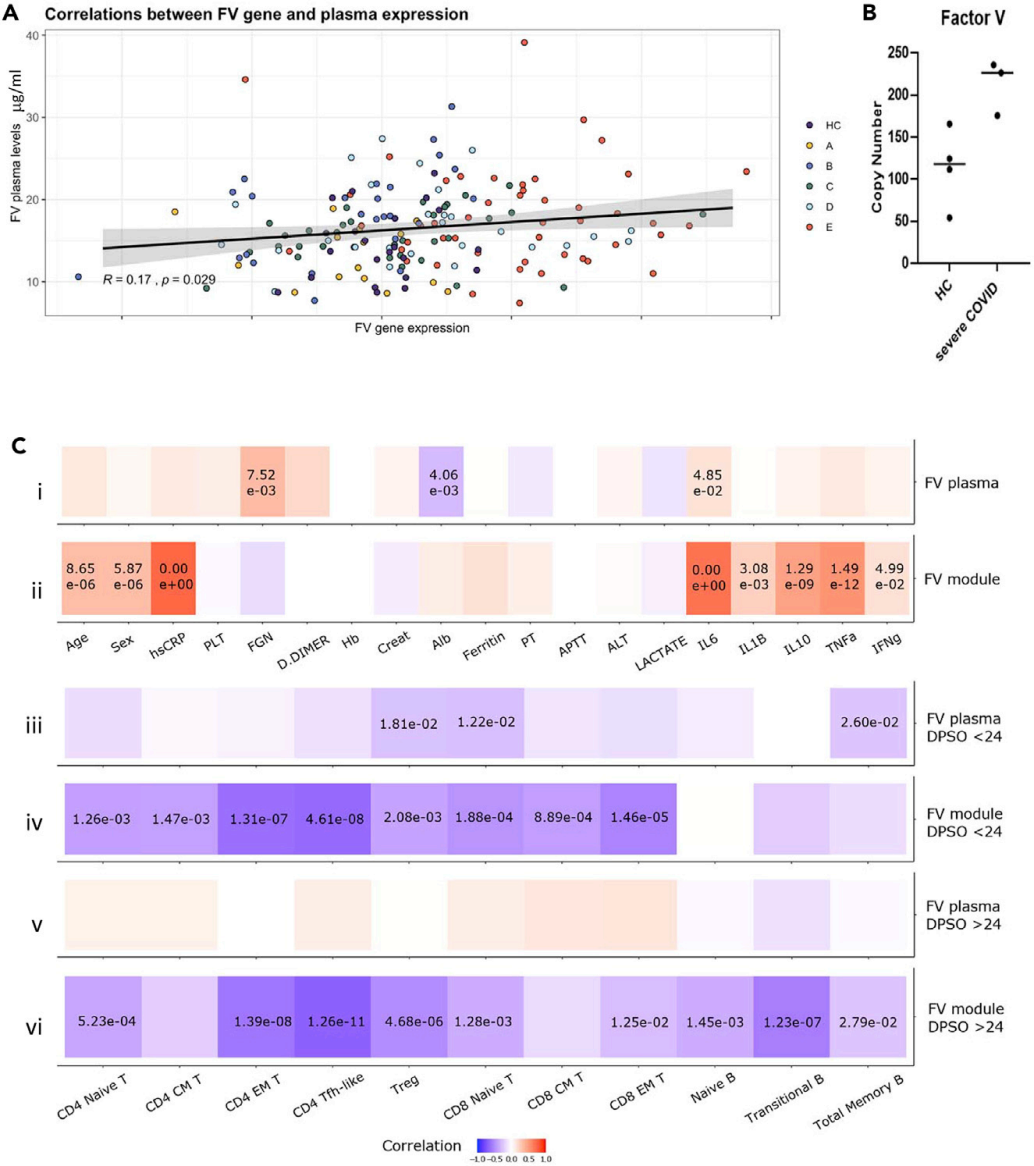
FV expression in COVID-19, and correlation with clinical and laboratory parameters (A) Correlation of plasma FV levels and peripheral blood cell FV gene expression in healthy controls and patients with COVID-19 (r = 0.17, p = 0.029). A, HCW screening asymptomatic; B, HCW screening symptomatic; C, hospitalized mild disease; D, hospitalized requiring oxygen; E, hospitalized, intensive care (see STAR Methods for details). (B) LC-MS measurement of FV in protein lysates of neutrophil extracts from healthy controls and patients with severe COVID-19. FV levels were significantly higher in neutrophil lysates from patients with severe COVID-19 compared to healthy (p = 0.025). (C) Correlation of plasma FV levels and FV module gene expression with predictors of disease severity and plasma protein levels (i-ii); or with B and T-cell counts (iii-vi). (i) FV plasma levels correlate with fibrinogen and IL6, whereas (ii) FV module gene expression correlated with predictors of disease severity (age, male gender, CRP) and increased plasma levels of IL6, IL1B, IL10, TNF and IFNγ. There was very little correlation between plasma factor V levels and T and B cell counts during the first 24 days after symptom onset (DPSO, iii) or after 24 days from the onset of symptoms (v). In contrast, FV module gene expression correlates with suppression of T-cell counts during the first 24 days post symptom onset (iv), and T and B cell counts after 24 days from the onset of symptoms (vi). p values are shown where significant. See also Figures S1 and S2.

There was a modest but statistically significant correlation between FV gene expression and FV protein expression (Figure 2A; p = 0.023, R = 0.17). Proteomic analysis showed significantly higher levels of FV in neutrophil lysates from patients with severe COVID-19 compared to healthy controls (Figure 2B).

We next explored whether FV expression correlated with biomarkers of disease severity. Consistent with our observation that FV gene expression increases in severe disease, we found that FV module gene expression correlates with predictors of disease severity (age, male gender, CRP) and increased plasma levels of IL6, IL10, interferon-γ and TNF, whereas FV plasma levels correlate only with fibrinogen and IL6 (Figures 2C i and 2C.ii). Furthermore, FV module gene expression correlates with suppression of T-cell counts, a marker of disease severity(Bergamaschi et al., 2021), during the first 24 days after symptom onset (Figure 2C iv), and T and B cell counts after 24 days from the onset of symptoms (Figure 2C vi). In contrast, there was very little correlation between plasma factor V levels and T and B cell counts during the first 24 days after symptom onset (Figure 2C iii) or after 24 days from the onset of symptoms (Figure 2C v).

### FV suppresses T-cell proliferation

**Figure 3:**
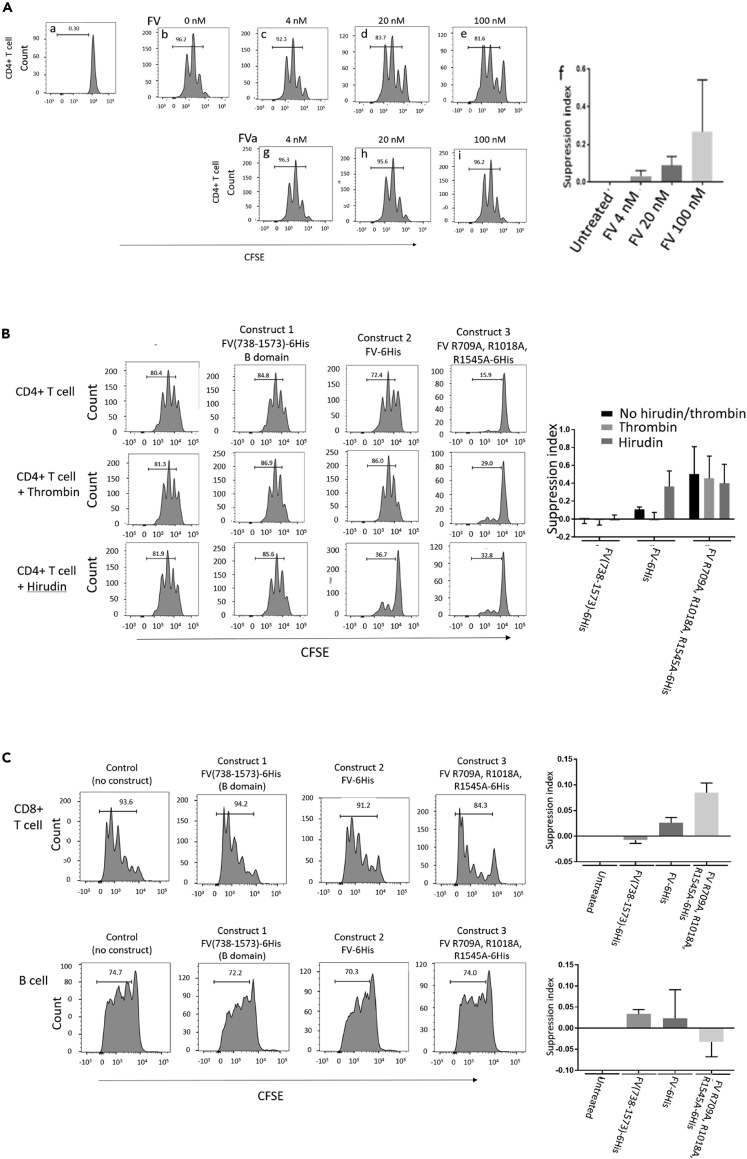
FV but not FVa suppresses T-cell proliferation *in vitro* (A) CD4+T conventional cells (Tcon) labeled with carboxyfluorescein diacetate succinimidyl ester (CFSE) (A), were stimulated with Dynabeads T-cell activator. Proliferation dilutes the dye, reducing fluorescence intensity with each cycle of cell division (B). Proliferation is inhibited in a concentration dependent manner by native Factor V (panels c – e), but not FVa (G – I).Data are representative of 10 healthy donors, and presented as mean and SEM (F). FV induced a significant suppression of T-cell proliferation in a concentration dependent manner: 4nM FV versus control p = 0.0077; 20 nM versus control p = 0.0003; 100 nM versus control p = 0.013. Bar chart shows mean and SEM. (B) CD4^+^ Tcon proliferation was not inhibited by recombinant FV B domain (Construct 1, 20 nM), and thrombin and hirudin had no effect on their own and in combination with construct 1. Recombinant full length Factor V (construct 2, 20 nM) inhibited CD4^+^ Tcon proliferation similar to native plasma derived Factor V, and its effect was prevented by thrombin (p = 0.036), while the effect of inhibition by mutated Factor V (construct 3, 20 nM) was not prevented by thrombin (p = 0.25). Data are representative of three healthy donors. Bar chart shows mean and SEM (C) CD8^+^T-cell proliferation was inhibited similarly by 20 nM construct 2 (p = 0.009) and construct 3 (p = 0.004), while B cell proliferation was not inhibited by any of the constructs (p = 0.1; p = 0.3; p = 0.6). Pro: proliferation. CD8^+^data are representative of four healthy donors, and B cell data are representative of three healthy donors. Bar chart shows mean and SEM.

The high level of expression of FV in Tregs in both patients and healthy controls (Figure 1C), and correlation between FV module expression and T-cell lymphopenia, led us to explore whether FV could suppress T-cell responses. To test this, CFSE-labeled conventional CD4^+^T-cells (Tcons) from healthy donors were polyclonally stimulated *in vitro* and proliferation was assessed by flow cytometry analysis of dye dilution. FV but not FVa suppressed proliferation of Tcons in a concentration dependent manner across the range 4–100 nM (Figure 3A). The IQR for plasma FV levels in healthy control subjects in the study was 32.2–51.2 nM, median 41.5 nM. To confirm that the suppressive effect was mediated by full length uncleaved FV we generated recombinant proteins from three FV constructs: (1) FV(738–1573)-6His (the B domain of FV); (2) FV-6His (full length Factor V); (3) Factor V R709A, R1018A, R1545A-6His (full length FV with Arginine thrombin cleavage sites mutated). Full length recombinant FV, but not a recombinant B domain, inhibited Tcon proliferation (Figure 3B). This effect was prevented by thrombin, and enhanced by the thrombin inhibitor hirudin. The cleavage-resistant recombinant FV was a potent inhibitor of Tcon proliferation. Recombinant full length FV and a cleavage-resistant recombinant FV also suppress CD8^+^T-cell proliferation, but not B cell proliferation (Figure 3C).

### FV is expressed by lung-infiltrating leukocytes in fatal COVID-19

**Figure 4:**
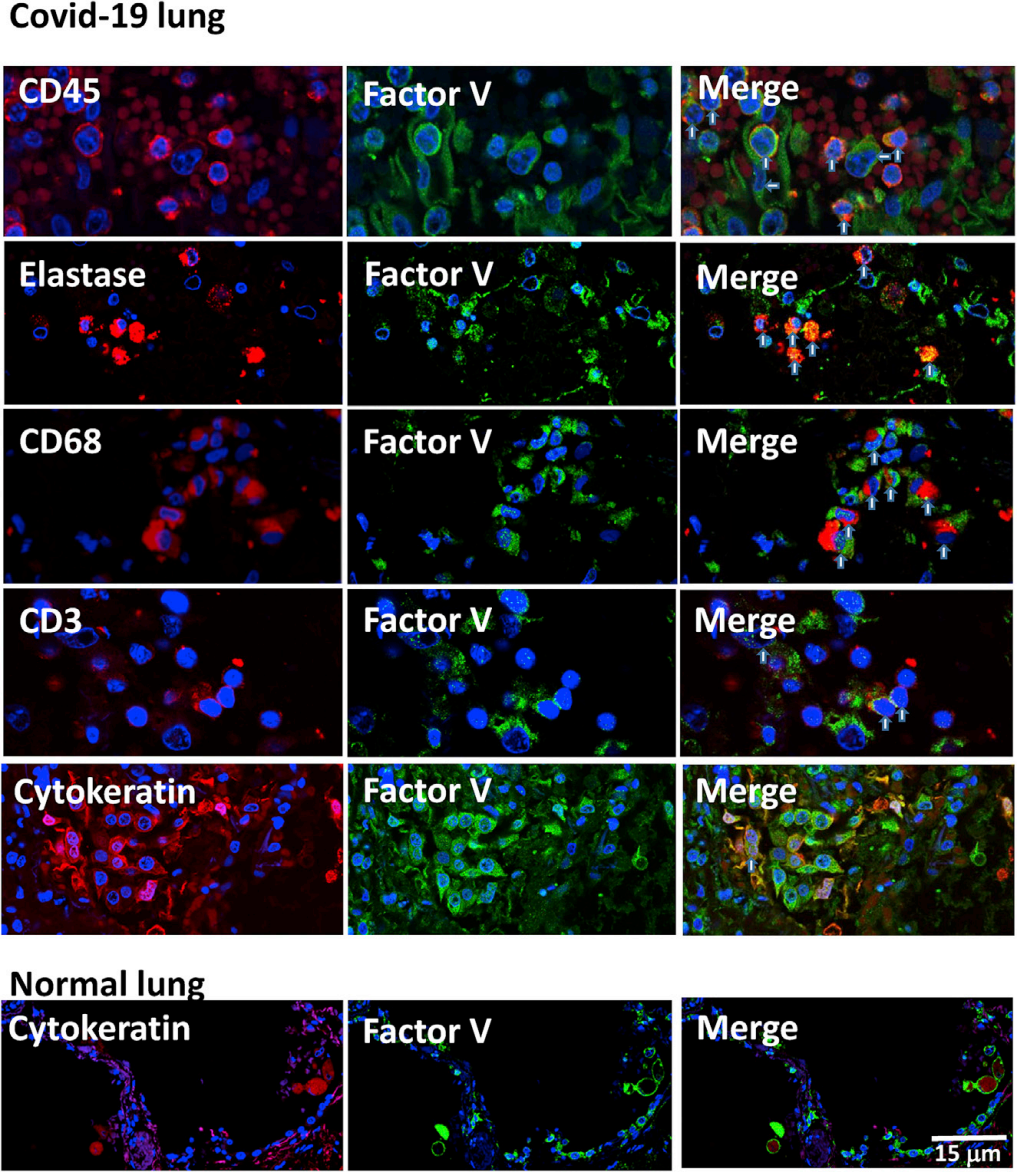
Factor V expression in COVID-19 lung tissue Immunostaining of COVID-19 lung tissue with anti-CD45 and anti-FV antibodies showed co-staining of CD45^+^ cells (red) with FV (green, vertical arrows on merged image), but also staining of some CD45^−^cells with FV (horizontal arrows on merged image). Co staining with FV (green) was also seen in cells expressing elastase (neutrophil marker, red), CD68 (monocyte lineage marker, red) and some CD3^+^ cells (red). Cells showing co-expression are identified with vertical arrows on the merged image. Co-staining for FV (green) was also seen in cells expressing cytokeratin (red) in COVID-19 lung tissue, where they form syncytia with bi-nucleate cells (arrow), and to a lesser extent in normal lung tissue. Scale bar 15 μm.

The significant correlation of lymphopenia with leukocyte expression of FV rather than with plasma levels prompted us to determine if production of the protein at a site of infection outside of the bloodstream might be occurring. To address this, we stained lung tissue from autopsies of four patients who died with COVID-19 lung disease. Strikingly, FV was increased within the lung parenchyma in fatal COVID-19 and was associated primarily with infiltrating neutrophils and monocytes (Figure 4). Some staining was also seen in a few CD3^+^T-cells and in alveolar epithelia. Control lung showed only low levels of FV in epithelial cells.

## Discussion

Our data show that circulating neutrophils, monocytes and Tregs are a source of increased FV in patients with severe COVID-19, both within the bloodstream and within lung parenchyma, and that FV but not thrombin activated FVa decreases T-cell proliferation *in vitro*. In patients with COVID-19 increased circulating blood cell FV expression correlates with T-cell lymphopenia, which can persist for at least ten weeks after infection.

There was a modest but statistically significant correlation between FV gene expression in circulating blood cells and plasma FV levels, but a role for leukocyte derived FV in regulating the immune response to SARS-CoV-2 is likely to occur following migration of leukocytes into the tissues, including secondary lymphoid organs from which circulating FV is normally excluded.

Broncho-alveolar lavage fluid from patients with severe COVID-19 has shown a predominance of neutrophils, monocytes/macrophages, and eosinophils with few lymphocytes, and in particular CD4 and CD8 T-cell lymphopenia, but an increase in the proportion of Tregs(Ronit et al., 2020). Furthermore lymphocytes were either absent or sparse in areas of SARs-CoV-2 pneumonia infiltrated by macrophages or neutrophils, or containing neutrophil extracellular traps (NETs) composed of neutrophil DNA, histones and granule-derived enzymes(Radermecker et al., 2020; Veras et al., 2020). Thus leukocyte derived FV may suppress local T-cell proliferation at sites of infection. In support of this we found a correlation between plasma FV levels and fibrinogen, biomarkers of hemostasis, whereas T-cell counts correlated with FV module gene expression in circulating leukocytes. We also found expression of FV in lung cells expressing cytokeratin, which in some areas appeared to be forming syncytia. Syncytial formation has been reported in lung tissue of patients with severe COVID-19(Braga et al., 2021; Bussani et al., 2020), and the presence of syncytia correlates with lymphopenia(Zhang et al., 2021). Overall these observations would be consistent with the liver being the predominant source of plasma FV, whereas leukocytes are an important source of FV in tissues. Local production of FV by leukocytes may also contribute to thrombosis, and neutrophil-rich macrothrombi have been found in heart autopsy tissue of patients who died from COVID-19(Johnson et al., 2022).

A number of variants in the *FV* gene are associated with abnormalities of coagulation. The commonest is FV Leiden, a G1691A variant resulting in a R506Q amino acid change, which reduces the inactivation of FVa by activated protein C(Van Cott et al., 2016). Heterozygous FV Leiden is found in about 5% of Caucasians, and homozygosity in about one in 5,000. It is unclear whether FV Leiden alters the immune inhibitory properties of FV, but FV Leiden heterozygosity may reduce the risk of developing sepsis from infection(Yan and Nelson, 2004).

East Texas bleeding disorder is caused by a rare variant at A2440G mutation in exon 13, predicting a S756G mutation in the B domain of FV, which causes upregulation of an alternatively spliced F5 transcript that results in a 250kDa isoform known as FV-short(Vincent et al., 2013). FV-short is thought to inhibit coagulation by forming a high-affinity complex with the coagulation inhibitor tissue factor pathway inhibitor-α (TFPIα). It is unclear whether FV-short has immunomodulatory properties, but we found TFPIα is increased in severe COVID-19 in concordance with FV (data not shown).

COVID-19 is associated with an increased risk of thromboembolism, but the role of anti-thrombotic agents remains unclear. In critically ill patients receiving organ support therapeutic dose heparin does not improve clinical outcomes or mortality, and may even cause harm(Goligher et al., 2021; Leentjens et al., 2021). Heparin binds to and activates anti-thrombin (Olson et al., 2010). LMWHs retain some anti-thrombin activity, but most of their activity is thought to result from inhibition of Factor Xa(Nutescu et al., 2016). Alternatives for VTE prophylaxis include warfarin and direct oral anticoagulants (DOACs). Warfarin depletes the reduced form of vitamin K that acts as a cofactor for gamma carboxylation of prothrombin, VII, IX and X, rendering them inactive(Wallin and Hutson, 2004). DOACs inhibit factor Xa or thrombin. The effect of anticoagulants on circulating factor V levels is unknown, but by inhibiting thrombin heparin reduces factor V activation, and could potentiate the T-cell suppressive effect of FV. In support of this there is evidence that heparin can directly suppress T-cell responses (Gorski et al., 1991). Increased FV expression by cells of the innate and adaptive immune systems may explain the lymphopenia seen in patients with severe Covid19. Heparin may potentiate suppression of the adaptive immune response by reducing FV activation.

### Limitations of the study

Our study is limited by recruitment of the cohorts from a single geographical region in the UK.

Sample size is critical when studying a heterogeneous disease, and COVID-19 falls into this category. A larger sample would allow more detailed analysis of the effect of demographic or disease related factors, and allow study of the effect of pro-thrombotic variants in the *FV* gene. In addition, our patients were recruited during the first wave of the pandemic, and a study examining infection with new SARS-CoV-2 strains with different virulence, and in vaccinated as well as unvaccinated patients would be interesting. Finally, it is unclear from this study whether increased leukocyte FV is specific for SARS-CoV-2, or a more general effect that could contribute to lymphopenia in viral infections.

## Consortia

Cambridge Institute of Therapeutic Immunology and Infectious Disease-National Institute for Health Research (CITIID-NIHR) COVID BioResource Collaboration

Stephen Baker, John R. Bradley, Patrick F. Chinnery, Daniel J. Cooper, Gordon Dougan, Ian Goodfellow, Ravindra K. Gupta; Nathalie Kingston, Paul J. Lehner, Paul A. Lyons, Nicholas J. Matheson, Caroline Saunders, Kenneth G. Smith, Charlotte Summers, James Thaventhiran, M. Estee Torok, Mark Toshner, Michael P. Weekes, Gisele Alvio, Sharon Baker, Areti Bermperi, Karen Brookes, Ashlea Bucke, Jo Calder, Laura Canna, Cherry Crucusio, Isabel Cruz, Rnalie deJesus, Katie Dempsey, Giovanni Di Stephano, Jason Domingo, Anne Elmer, Julie Harris, Sarah Hewitt, Heather Jones, Sherly Jose, Jane Kennet, Yvonne King, Jenny Kourampa, Emily Li, Caroline McMahon, Anne Meadows, Vivien Mendoza, Criona O'Brien, Charmain Ocaya, Ciro Pascuale, Marlyn Perales, Jane Price, Rebecca Rastall, Carla Ribeiro, Jane Rowlands, Valentina Ruffolo, Hugo Tordesillas, Phoebe Vargas, Bensi Vergese, Laura Watson, Jieniean Worsley, Julie-Ann Zerrudo, Laura Bergamaschi, Ariana Betancourt, Georgie Bower, Ben Bullman, Chiara Cossetti, Aloka DeSa, Benjamin Dunore, Maddie Epping, Stuart Fawke, Stefan Gräf, Richard Grenfell, Andrew Hinch, Josh Hodgson, Christopher Huang, Oisin Huhn, Kelvin Hunter, Isobel Jarvis, Emma Jones, Maša Josipović, Ekaterina Legchenko, Daniel Lewis, Joe Marsden, Jennifer Martin, Federica Mescia, Francesca Nice, Ciara O'Donnell, Ommar Omarjee, Marianne Perera, Linda Pointon, Nicole Pond, Nathan Richoz, Nika Romashova, Natalia Savoinykh, Rahul Sharma, Joy Shih, Mateusz Strezlecki, Rachel Sutcliffe, Tobias Tilly, Zhen Tong, Carmen Treacy, Lorinda Turner, Jennifer Wood, Marta Wylot, John Allison, Hila Apelbaum, Alessandra Barreto Da Silva, Heather Biggs, Helen Butcher, Daniela Caputo, Matt Chandler, Debbie Clapham-Riley, Anne-Maree Dean, Eleanor Dewhurst, Rose Eichenberger, Christian Fernandez, Anita Furlong, Anne George, Barbara Graves, Jennifer Gray, Sabine Hein, Tasmin Ivers, Emma Le Gresley, Rachel Linger, Mary Kasanicki, Sarah Meloy, Alexei Moulton, Francesca Muldoon, Nigel Ovington, Sofia Papadia, Christopher Penkett, Isabel Phelan, Venkatesh Ranganath, Roxana Paraschiv, Jennifer Sambrook, Katherine Schon, Hannah Stark, Paul Townsend, Julie von Ziegenweidt, Jennifer Webster, Sabrina Rossi, Mayurun Selvan, Sarah Spencer, Cissy Yong, Petra Polgarova, Sarah Caddy, Laura Caller, Yasmin Chaudhry, Martin Curran, Theresa Feltwell, Iliana Georgana, Grant Hall, William Hamilton, Myra Hosmillo, Charlotte Houldcroft, Rhys Izuagbe, Aminu Jahun, Fahad Khokhar, Anna Kovalenko, Luke Meredith, Surendra Parmar, Malte Pinckert, Anna Yakovleva

## STAR★Methods

### Key resources table

**Table:** 

REAGENT or RESOURCE	SOURCE	IDENTIFIER
**Antibodies**		

Rabbit anti human FV	Abcam	Cat#ab234849
Mouse anti human CD45	Agilent DAKO	Cat#M070101-2, Clones 2B11 + PD7/26; RRID:AB_2750582
Mouse anti human Elastase	R&D systems	Cat#MAB91671, Clone 950317
Mouse anti human CD68	Abcam	Cat#ab199000, Clones KP1 + C68/684
Mouse anti human CD3	Abcam	Cat#ab17143, Clone F7.2.38; RRID:AB_782094
Mouse anti human cytokeratin	Abcam	Cat#ab27988, Clone AE1/AE3; RRID:AB_10717335

**Biological samples**		

Covid patient lung tissue	Cambridge University Hospital Tissue Bank, https://www.cuh.nhs.uk/our-research/research-facilities/tissue-bank	Cat#G14678

**Chemicals, peptides, and recombinant proteins**		

Native human FV	Invitrogen	Cat#RP-43126
Native human FVa	Invitrogen	Cat#RP-43100
Thrombin	Invitrogen	Cat#RP-43128
Hirudin	Sigma-Aldrich	H0393; CAS:8001-27-2
Recombinant FV B domain (aa710-1545)	Peak Proteins	http://peakproteins.com
Recombinant FV (aa 1 - 2224)	Peak Proteins	http://peakproteins.com
Recombinant FV (aa 1 - 2224) [R709A, R1018A, R1545A]	Peak Proteins	http://peakproteins.com

**Critical commercial assays**		

CFSE labeling kit	Invitrogen	Cat#C34554
FV ELISA kit	Abcam	Cat#137976
Human B cell expansion kit	R&D system	Cat#CDK005
CD4 Tcell isolation kit	Miltenyi	Cat#130-091-301
CD8 MicroBeads	Miltenyi	Cat#130-045-201
Pan B cell isolation kit	Miltenyi	Cat#130-101-638

**Deposited data**		

Whole blood RNAseq data	Bergamaschi et al., 2021, https://doi.org/10.1016/j.immuni.2021.05.010, and this paper	https://ega-archive.org/search-results.php?query=EGAS00001005332
scRNAseq data	Stephenson et al., 2021, https://doi.org/10.1038/s41591-021-01329-2, and this paper	https://www.ebi.ac.uk/arrayexpress/experiments/E-MTAB-10026/
Clinical and demographic data	Bergamaschi et al., 2021, https://doi.org/10.1016/j.immuni.2021.05.010, and this paper	Table S1: Clinical features of study participants, stratified by group A-E; https://ars.els-cdn.com/content/image/1-s2.0-S1074761321002168-mmc1.pdfhttps://www.covid19cellatlas.org/patient/citiid/

**Experimental models: Cell lines**		

Human CD4^+^ T cells	NHS blood and transplant service	https://www.nhsbt.nhs.uk/
Human CD8^+^ T cells	NHS blood and transplant service	https://www.nhsbt.nhs.uk/
Human B cells	NHS blood and transplant service	https://www.nhsbt.nhs.uk/

**Software and algorithms**		

R	R Core Team, 2015	N/A
Flowjo_v10.0.8	BD Biosciences	https://www.flowjo.com/
GraphPad Prism 9	Dr. Harvey Motulsky	https://www.graphpad.com/scientific-software/prism/
Gene set enrichment analysis (GSEA)	Broad Institute	https://www.gsea-msigdb.org/gsea/index.jsp
GO enrichment analysis	Gene ontology consortium	http://geneontology.org/docs/go-enrichment-analysis/
Enrichr	Ma’ayan lab	https://maayanlab.cloud/Enrichr/#about
FastQC v.0.11.8	Babraham Bioinformatics, UK	https://www.bioinformatics.babraham.ac.uk/projects/fastqc/
Trim_galore v.0.6.4	Babraham Bioinformatics, UK	https://www.bioinformatics.babraham.ac.uk/projects/trim_galore/
BBMap v.38.67	BBMap - Bushnell B	https://sourceforge.net/projects/bbmap/
Blueprint epigenome	Blueprint consortium	https://www.blueprint-epigenome.eu/
Specronaut 14	Biognosys	https://biognosys.com/software/spectronaut/

### Resource availability

#### Lead contact

Further information and requests for resources and reagents should be directed to and will be fulfilled by the lead contact, John Bradley (jrb1000@cam.ac.uk).

#### Materials availability

• This study did not generate new unique reagents.

### Experimental model and subject details

#### Healthy volunteers and patients

Healthy donor blood samples were obtained from the NIHR BioResource Centre Cambridge and leukapheresis samples from the National Health Service Blood and Transfusion services (NHSBT, Cambridge) with written informed consent of donors and approval of the National Research Ethics Committee and Health Research Authority. Healthcare workers and patients with COVID-19 confirmed by Nucleic acid amplification testing(Collier et al., 2020) of nasopharyngeal swabs for SARS-CoV-2 were consented to the NIHR COVID-19 cohort of the NIHR BioResource (https://bioresource.nihr.ac.uk/using-our-bioresource/our-cohorts/covid-19-bioresource/) between 31/3/2020 and 20/7/2020 with approval of the National Research Ethics Committee and Health Research Authority (East of England – Cambridge Central Research Ethics Committee (“NIHR BioResource” REC ref 17/EE/0025). These included patients presenting to Cambridge University Hospitals and Royal Papworth Hospital, together with asymptomatic or symptomatic healthcare workers (HCWs) undergoing routine screening. Timing of samples refers to the number of days after onset of symptoms or a positive swab in asymptomatic healthcare workers (HCW). Hospitalised patients underwent a venous thromboembolism risk assessment and received prophylactic dalteparin if there was low bleeding risk. From 1 May 2020 patients were discharged with 2 weeks of prophylactic dalteparin following a risk assessment.

The demographics, clinical and laboratory assessments of the participants have been previously described(Bergamaschi et al., 2021). 18 asymptomatic healthcare workers (group A) were 22.2% male and had a mean (SD) age of 32.9 (12.7); 40 symptomatic healthcare workers (group B) were 22.5% male and had a mean (SD) age of 36.0 (11.8); 46 hospitalised patients with mild disease (group C) were 54,3% male and had a mean (SD) age of 58.0 (16.9); 37 hospitalised patients requiring oxygen (group D) were 64.9% male and had a mean (SD) age of 64.4 (15.1); 60 hospitalised patients requiring intensive care (group E) were 75.0% male and had a mean (SD) age of 57.0 (14.9.

For neutrophil proteomic studies peripheral venous blood was taken from healthy volunteers (age 25–60, one female) with written informed and approval of the University of Edinburgh Centre for Inflammation Research Blood Resource Management Committee. The collection of peripheral venous blood from patients (age 41–56, one female) with COVID-19 was approved by Scotland A Research Ethics Committee. Patients receiving ventilation in intensive care at the Royal Infirmary of Edinburgh were recruited between April and August 2020, with informed consent obtained by proxy.

Post mortem tissue was collected under the Use of Post-Mortem Examination Tissue and Organs for Research in Patients with COVID-19 - COPE Study with approval of the National Research Ethics Committee and Health Research Authority.

#### Peripheral blood cell isolation and culture

PBMCs were isolated from leukapheresis samples and whole blood by polysucrose density gradient centrifugation (Ficoll-Paque, GE Healthcare Life Sciences, UK). Leukapheresis samples are de-identified and age and gender of donor is not known. Cells were grown at 37°C in RPMI media supplemented with 10% human AB serum.

Neutrophils were isolated from blood by dextran sedimentation and discontinuous Percoll gradients(Reyes et al., 2021). Up to 80 mL of whole blood was collected into citrate tubes and centrifuged at 300 × g (acceleration 5, deceleration 5) for 20 min and the platelet-rich plasma layer removed. Erythrocyte sedimentation and leukocyte-rich plasma were obtained by incubating the remaining contents in the tube with 6 mL of 6% Dextran 500 in saline and final volume adjusted to 50 mL with 0.9% NaCl for at least 20 min at room temperature. The leukocyte-rich portion was centrifuged at 350 × g (acceleration 5, deceleration 5) for 6 min, with the pellet resuspended in 3 mL of 49.5% Percoll (GE Healthcare) and overlayed onto 61.2% Percoll and 72.9% Percoll. Gradients were centrifuged at 720 × g (acceleration 1, deceleration 0) for 20 min to obtain neutrophil and PBMC layers.

To prepare proteomic samples neutrophils were centrifuged at 300 × g for 5minat 4°C and resuspended in 7 mL of 0.2% NaCl (w/v in H _2_O) for 5minat room temperature and topped up with 7 mL of 1.6% NaCl (w/v in H _2_O). Cells were washed twice in Dulbecco’s phosphate-buffered saline (DPBS; Thermo Fisher), pelleted at 300 × g for 5minat 4°C and resuspended in 372 μL of freshly made 5% sodium dodecyl sulfate (SDS, BioRad) lysis buffer and vortexed. Samples were then heat denatured in a heat block for 5minat 100°C and stored at −80°C. Cell lysates were thawed and tris(2-carboxyethyl) phosphine hydrochloride (TCEP) and triethylammonium bicarbonate (TEAB) were added to a final concentration of 10 and 50 mM, respectively. Lysates were shaken at 500rpmat 22°C for 5 min before being incubated at 98°C for 5 min. Samples were allowed to cool and were then sonicated with a BioRuptor (30 cycles: 30 s on and 30 s off). Tubes were centrifuged at 17,000 × g to collect the cell lysate and 1 mL of benzonase (27.8 units) was added to each sample and samples incubated at 37°C for 15 min. Samples were then alkylated with addition of 20 mM iodoacetamide for 1hat 22°C in the dark. Protein lysates were processed for mass spectrometry using ProTifi s-trap spin columns following the manufacturer’s instructions. Lysates were digested with Trypsin at a ratio 1:20 (protein:enzyme) in 50 mM ammonium bicarbonate. Peptides were eluted from s-trap columns by sequentially adding 80 mL of 50 mM ammonium bicarbonate followed by 80 mL of 0.2% formic acid with a final elution using 80 mL of 50% acetonitrile + 0.2% formic acid.

### Method details

#### Analysis of neutrophil lysates

Analysis of neutrophil lysates was performed using liquid chromatography mass spectrometry (LC-MS)-based proteomics(Reyes et al., 2021). For each sample, 2 mg of peptide was analysed on a Q-Exactive-HF-X (Thermo Scientific) mass spectrometer coupled with a Dionex Ultimate 3000 RS (Thermo Scientific). LC buffers were the following: buffer A (0.1% formic acid in Milli-Q water (v/v)) and buffer B (80% acetonitrile and 0.1% formic acid in Milli-Q water (v/v)). 2 μg aliquot of each sample were loaded at 15 μL/min onto a trap column (100 μm × 2 cm, PepMap nanoViper C18 column, 5 μm, 100 Å, Thermo Scientific) equilibrated in 0.1% trifluoroacetic acid (TFA). The trap column was washed for 3minat the same flow rate with 0.1% TFA then switched in-line with a Thermo Scientific, resolving C18 column (75 μm × 50 cm, PepMap RSLC C18 column, 2 μm, 100 Å). The peptides were eluted from the column at a constant flow rate of 300 nL/min with a linear gradient from 3% buffer B to 6% buffer B in 5 min, then from 6% buffer B to 35% buffer B in 115 min, and finally to 80% buffer B within 7 min. The column was then washed with 80% buffer B for 4 min and re-equilibrated in 3% buffer B for 15 min. Two blanks were run between each sample to reduce carry-over. The column was kept at a constant temperature of 50°Cat all times.

#### Whole blood bulk mRNA-Seq

##### Library preparation and RNA-Seq processing

RNA was quantified using RNA HS assay on the Qubit, and libraries prepared using the SMARTer® Stranded Total RNA-Seq it v2 - Pico Input Mammalian kit (Takara) with 10ng of RNA as starting input. Library quality and quantity were validated by capillary electrophoresis on an Agilent 4200 TapeStation. Libraries were pooled at equimolar concentrations, and paired-end sequenced (75bp) across 4 lanes of a Hiseq4000 instrument (Illumina) to achieve 10 million reads per samples.

##### Reads mapping and quantification

The quality of raw reads was assessed using FastQC (http://www.bioinformatics.babraham.ac.uk/projects/fastqc/). SMARTer adaptors were trimmed, along with sequencing calls with a Phred score below 24 using Trim_galore v.0.6.4 (http://www.bioinformatics.babraham.ac.uk/projects/trim_galore/.) Residual rRNA reads were depleted in silico using BBSplit (https://github.com/BioInfoTools/BBMap/blob/master/sh/bbsplit.sh).

Alignment was performed using HISAT2 v.2.1.0(Kim et al., 2019) against the GRCh38 genome build achieving a more than 95% alignment rate. A count matrix was generated in R using featureCounts (Rsubreads - packages) and converted into a DGEList (EdgeR package), for downstream analysis.

##### Downstream analysis

Downstream analysis was performed in R. Counts were filtered using filterByExpr (EdgeR package) with a gene count threshold of 10CPM and the minimum number of samples set as the size of the smallest disease group. Library counts were normalised using calcNormFactors (EdgeR package) using the method ‘weighted trimmed mean of M-values’. The function ‘voom’ (limma package) was applied to the data to estimate the mean-variance relationship, allowing adjustment for heteroscedasticity.

#### scRNAseq of PBMCs

##### Healthy volunteers and patients

scRNAseqdata is included for 47 individuals recruited in Cambridge for whom FV data is available. These volunteers form part of a larger cohort of 130 volunteers in whom scRNAseq was performed(Stephenson et al., 2021). The clusters of cells in the Cambridge cohort have been annotated according to the sets of marker genes and proteins previously reported. Differential gene expression analyses across patients within each cell subtype have been previously reported for the larger cohort(Stephenson et al., 2021).

##### Sample pre-processing

Purified PBMCs were thawed at 37°C, transferred to a 50 mL tube and 10 volumes of pre-warmed thawing media (IMDM –Gibco 12440-053-, 50% FCS (not heat inactivated) – Panbiotech P40-37500, 0.1 mg/mL DNaseI– Worthington LS002139) were added slowly and dropwise, followed by centrifugation at 500*g* for 5 min. The pellet was resuspended in 1 mL of FACS buffer (PBS-Sigma D8537-500mL-, 3% Heat Inactivated FCS) and viability of each sample was assessed by counting in an improved Neubauer chamber using Trypan blue. Pools of 4 samples were generated by combining 0.5 million live cells per individual (2 million live cells total). The pools were washed twice in FACS buffer (10 and 2 mL, respectively) followed by centrifugation for 5minat 500 g. The pellet was then resuspended in 35 μL of FACS buffer and the viability of each pool was assessed.

##### Antibody staining

Half a million viable cells resuspened in 25 μL of FACS buffer and incubated with 2.5 μL of Human TruStain FcX™ Fc Blocking Reagent (BioLegend 422302) for 10minat 4°C. The TotalSeq-C™ antibody cocktail (BioLegend 99813) was centrifuged at 14,000gat 4°C for 1 min, resuspended in 52 μL of FACS buffer, incubated at room temperature for 5 min and centrifuged at 14,000gat 4°C for 10 min 25 μL were subsequently added to each sample pool and incubated for 30minat 4°C in the dark. Pools were washed 3 times with 27 vol (1.4 mL) of FACS buffer, followed by centrifugation at 500*g* for 5 min. The pellet was resuspended in 62.5 μL of 1x PBS + 0.04% BSA (Ambion, #AM2616), filtered through a 40 μm cell strainer (Flowmi H13680-0040) and viability of each sample pool was assessed.

##### 10XGenomics droplet single-cell RNA-sequencing

50,000 live cells (up to a maximum of 60,000 total cells) for each pool were processed using Single Cell VDJ 5′ version 1.1 (1000020) together with Single Cell 5′ Feature Barcode library kit (1000080), Single Cell V(D)J Enrichment Kit, Human B Cells (1000016) and Single Cell V(D)J Enrichment Kit, Human T Cells (1000005) from 10X Genomics following manufacturer’s recommendations. The samples were subjected to 12 cycles of cDNA amplification and 8 cycles for the protein library construction. The rest of libraries were processed as indicated by the manufacturer.

Libraries were pooled per sample using the ratio 9:2.4:1:0.6 for gene expression, feature barcoding, TCR enriched and BCR enriched libraries.

Samples were sequenced in Illumina NovaSeq6000 sequencer machine using S1 flowcells.

##### Single-cell RNA-sequencing processing, demultiplexing and quality control

Multiplexed 10X scRNA-seq GEX libraries were aligned to the human genome, reads deduplicated, and UMIs quantified using Cellranger v4.0Drop utilising the hg38 genome reference sequence. Gene expression count matrices of genes by droplets were generated separately for each multiplex pool of 4 donors. Within each sample, single-cells were delineated from background empty droplets using emptyDrops implemented in the Bioconductor packages DropletUtils(Lun et al., 2016, 2019) with a background UMI threshold of 100. Cell

Libraries were demultiplexed and single-cells were assigned to one of the 4 constituent donors in each library. Single-cell genotypes were derived from the sequencing data using CellSNP (https://github.com/single-cell-genetics/cellSNP), and best-matching donors were assigned by comparing the single-cell genotypes to the germ-line genotyping for each donor using vireo(Huang et al., 2019). Cells that could not be assigned confidently to a single donor were removeprior to analyses. Furthermore, cells that were confidently assigned to multiple donors were classed as doublets and removed prior to down-stream analyses.

Poor quality cells were removed based on an excess of mitochondrial expressed genes, defined as > 7% of all UMIs in each single-cell. Sparsely-sequenced cells were also removed where the total UMI count for a cell was <1000. Deconvolution normalisation factors were then estimated for all cells across all samples combined, prior to log10 transformation with a pseudocount (+1), as implemented in scran(Lun et al., 2016).

The probability of being a doublet was estimated for each cell per sample using the “doubletCells” function in scran based on highly variable genes (HVGs). Highly variable genes (HVG) were defined across all single-cells by modelling the mean-variance relationship across genes using a loess fit, as implemented in the modelGeneVar function in scran. Next, we used “cluster_walktrap”https://arxiv.org/abs/physics/0512106) on the shared nearest-neighbour (SNN)-graph that was computed on HVGs to form highly resolved clusters per sample. Per-sample clusters with either a median doublet score greater than the median + 2.5 x MAD or clusters containing more than the median + 2.5 MAD genotype doublets were tagged as doublets. This was followed by a second round of highly-resolved clustering across the whole data set, in which again cells belonging to clusters with a high proportion (>60%) of cells previously labelled as doublets were also defined as doublets.

#### Collection and analysis of post mortem tissue

Post mortem lung tissue (n = 4) was collected by Cambridge University Hospitals tissue bank under the Use of Post-Mortem Examination Tissue and Organs for Research in Patients with COVID-19 - COPE Study with approval of the National Research Ethics Committee and Health Research Authority (research ethics committee reference 20/WM/0270). De-paraffinized sections were exposed to high-pressure antigen retrieval before incubation with anti-FV and cell specific markers overnight (4°C). This was followed by specified-specific secondary antibody-conjugated to Alexa flour-488 or Northern light−557 plus Hoechst 333342 (1 μg/mL) for nuclei detection for 1 h (1:100), mounted in VectaShield (Vector Laboratories) before viewing on a TCS-SPE CLSM (Leica Microsystems).Isotype-specific sera was used as a negative control. Image for each fluorophore was acquired sequentially using the same constant acquisition time and settings rather than simultaneously to avoid crosstalk between channels.

Antibodies used were rabbit anti-human FV (Abcam, ab234849), mouse anti-human CD45 (DAKO, 2B11 + PD7/26), mouse anti-human neutrophil elastase (RnD system, 950317), mouse anti-human CD68 (Abcam, KP1+684), mouse anti-human CD3 (Abcam, F7.2.38); replicate experiments were also performed with mouse anti-human FV (Cambridge Bioscience, AHV-5102), rabbit anti-human MPO (DAKO, A0398), rabbit anti-human CD68 panel (Abcam, ab254013), rabbit anti-human CD3 (Abcam, ab16669).

#### T-cell *in vitro* expansion assay

B cell proliferation kit (R&D system, Abingdon, UK). BD CellFix (BD Biosciences, Oxford, UK). Native human FV, FVa, Thrombin, Dynabeads Human T-cell activator, and CFSE kits (Thermofisher Scientific, Loughborough, UK). Human Factor V ELISA Kit (ab137976, Abcam, Cambridge, UK). Unless otherwise indicated, all reagents were from Sigma-Aldrich Company Ltd (Dorset, UK).

CD4 T cell, CD8 T cell and B cell were isolated using Miltenyi isolation kits (Miltenyi Biotec, Surrey, UK) following the manufacturer’s instruction. Cells were stained with CFSE using CFSE kits (Thermofisher Scientific, Loughborough, UK) following the manufacturer’s instruction. 5 × 10^4^ labelled T cons were mixed with Dynabeads Human T cell activator CD3/CD28 (Thermofisher Scientific, Loughborough, UK) at cell-to-beads ratio of 2 to 1 and incubated at 37°C. Cells incubated with native human FV, FVa, FV constructs at the concentrations indicated, thrombin (5 μg/mL), and hirudin (5 iU/ml) were collected and analysed using a BD Fortessa flow cytometer after 4 or 5 days.

#### Expression of FV constructs

Three constructs comprising (1) FV B domain ((aa710-1545), (2) full length FV (aa 1 - 2224) and (3) [R709A, R1018A, R1545A]FV aa1-2224 were sub-cloned into a proprietary vector for the HEK293-6E system by Peak Proteins. All sequences contained a C-terminal 6His tag to facilitate purification. Cells were transfected at a 500 mL scale for each construct, media harvested 5-6 days after transfection and protein purified using a combination of Ni affinity and size exclusion chromatography and if required ion exchange. Purified proteins were analysed by reducing and non-reducing SDS-PAGE, A280 to determine concentration, size exclusion and mass spectrometry to confirm identity.

### Quantification and statistical analysis

Statistical analyses were conducted using custom scripts in R. Absolute cell counts (cells/uL) were offset by +1 to allow subsequent log2 transformation of zero counts. Where shown, time measures represent time from symptom onset (for severity groups B, C, D and E) or first positive COVID-19 swab (group A). Unless otherwise specified, longitudinally collected data was grouped by bins of 7 or 12 days. Pairwise statistical comparison of absolute cell counts, CRP or serum measures between individuals in a given severity group at a given time bin and HCs, or between severity groups, was conducted by Wilcoxon test unless otherwise specified. For analyses involving repeated-measures, false discovery rate corrected (Benjamini & Hochberg) p value were reported. For individuals sampled more than once within a given time bin, data from the earliest blood collection was used.

#### Weighted gene co-expression network and gene enrichment analysis

The weighted gene co-expression network analysis (WGCNA) package in R overcomes the problem of multiple testing by grouping co-correlated genes into modules and relating them to clinic traits. Modules are not comprised of*a priori* defined gene sets but rather are generated from unsupervised clustering. The eigengene of the module is then correlated with the sample traits and significance determined. A signed adjacency matrix was generated, and a soft thresholding power chosen to impose approximate scale-free topology. Modules identified from the topological overlap matrix had a specified minimum module size of 30. Significance of correlation between a clinical trait and a modular eigengene was assessed using linear regression with Bonferroni adjustment to correct for multiple testing. Modules were annotated using Enrichr and Genemania. Genes with high connectivity termed “hub genes” were identified based on a module membership of 0.8 or above and were selected to have a correlation with the trait of interest ≥0.8.

Gene enrichment analysis was performed using the Enrichr and GO enrichment analysis tools.

##### Correlation

The relationships between multiple features were quantified using Pearson’s correlation (Hmisc package) and visualized with corrplot.

##### Mixed effects model

Longitudinal mixed modelling of factor V module eigenvalue changes over time $\left(y_{ij}\right)$ was conducted using the nlme package in R, including time $\left(t_{ij}\right)$ with a quadratic trend and disease severity $\left(X_{j}\right)$ as fixed effects, and sampled individuals as random effects $\left(u_{j}\right)\text{.}$

#### Proteomic data analysis

Data were analysed with Spectronaut 14 using the direct data-independent acquisition option 27 (Skyline, MacCoss Lab Software is a freely available alternative). Cleavage Rules were set to Trypsin/P, Peptide maximum length was set to 52 amino acids, Peptide minimum length was set to 7 amino acids and Missed Cleavages set to 2. Calibration Mode was set to Automatic. Search criteria included carbamidomethylation of cysteine as a fixed modification, as well as oxidation of methionine, deamidation of asparagine and glutamine and acetylation (protein N-terminus) as variable modifications. The false discovery rate threshold was set to 1% Q-value at both the Precursor and Protein level. The single hit definition was to Stripped sequence. Data were searched against the human SwissProt database (July 2020) and included isoforms. The Major Group Quantity was set to the Sum of peptide quantity and the Minor Group Quantity was set to the Sum of the precursor quantity; Cross Run Normalization was disabled. Fold changes and p values were calculated in R utilising the bioconductor package LIMMA version 3.7 28. The Q-values provided were generated in R using the “qvalue” package version 2.10.0. Estimates of protein copy numbers per cell were calculated using the histone ruler method 29. The mass of individual proteins was estimated using the following formula: CN × MW/NA = protein mass (g cell −1), where CN is the protein copy number, MW is the protein molecular weight (in Da) and NA is Avogadro’s Constant.

#### Single-cell RNA-sequencing clustering and annotation

Highly variable genes (HVG) were defined across all single-cells that passed QC and that were successfully assigned to a donor individual, by modelling the mean-variance relationship across genes using a loess fit, as implemented in the modelGeneVar function in scran. HVGs (FDR 1%) were used as input to estimate the first 50 principal components using the IRLBA implementation in the R package irlba. Single-cell clusters were computed by first constructing a k-nearest neighbour graph (k = 20). Cells were broadly grouped into discrete clusters based on the Walktrap community detection algorithm (https://arxiv.org/abs/physics/0512106), using the kNN-graph as input.

Each cluster was annotated into one of 7 broad categories based on the expression of canonical marker genes for each: CD4 T-cell, CD8 T-cell, NK cell, Monocyte, Plasma cell, B cell and dendritic cell. We then subset the cells within each category and we re-computed HVGs, PCA, k-NN graph and clusters as described above.

#### T-cell proliferation assay

T and B cell flow cytometry data of were analysed using FlowJo (Tree Star, USA). Graphs and statistics were generated using GraphPad Prism software. Results were presented as mean ± s.e.m.as indicated. Differences between two groups were compared using two-tailed student’s t-test. Suppression index was calculated as the ratio between decreased percentage of proliferation and the total percentage of proliferation of the cells.

## Data Availability

• scRNAseqdata is available through ArrayExpress. The DOI is listed in the key resources table. Whole blood RNAseq is available at the European Genome Archive. The DOI is listed in the key resources table. • De-identified human/patient data are publicly available as of the date of publication. DOI and accession numbers are listed in the key resources table. • This paper does not report original code. • Any additional information required to reanalyze the data reported in this paper is available from the lead contact upon request.
