# Supplementary material for: Coagulation factor V is a T-cell inhibitor expressed by leukocytes in COVID-19
Source: iScience. 2022 Feb 22;25(3):103971. doi: 10.1016/j.isci.2022.103971 (PMC8863325; doi:10.1016/j.isci.2022.103971)
Supplement: Document S1. Figures S1–S3 and Tables S1 [file mmc1.pdf]

iScience, Volume *25*

Supplemental information

Coagulation factor V is a T-cell inhibitor expressed by leukocytes in COVID-19

Jun Wang, Prasanti Kotagiri, Paul A. Lyons, Rafia S. Al-Lamki, Federica Mescia, Laura Bergamaschi, Lorinda Turner, Michael D. Morgan, Fernando J. Calero-Nieto, Karsten Bach, Nicole Mende, Nicola K. Wilson, Emily R. Watts, Cambridge Institute of Therapeutic Immunology and Infectious Disease-National Institute of Health Research (CITIID-NIHR) Covid BioResource Collaboration, Patrick H. Maxwell, Patrick F. Chinnery, Nathalie Kingston, Sofia Papadia, Kathleen E. Stirrups, Neil Walker, Ravindra K. Gupta, David K. Menon, Kieren Allinson, Sarah J. Aitken, Mark Toshner, Michael P. Weekes, James A. Nathan, Sarah R. Walmsley, Willem H. Ouwehand, Mary Kasanicki, Berthold Göttgens, John C. Marioni, Kenneth G.C. Smith, Jordan S. Pober, and John R. Bradley

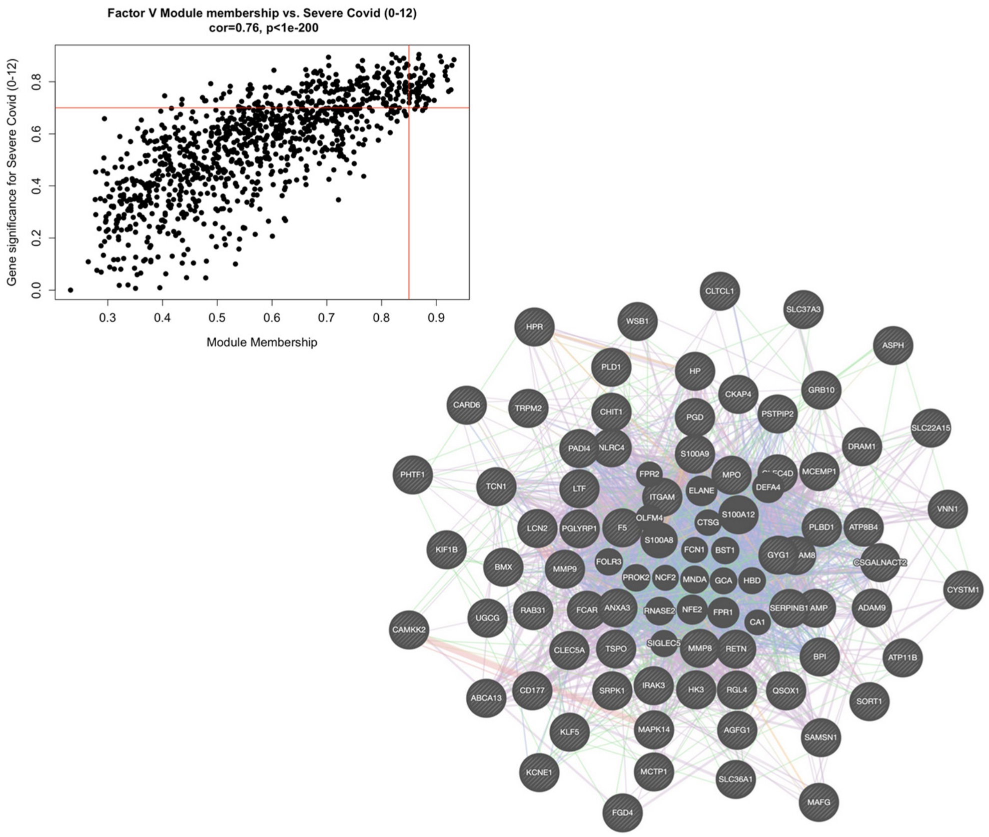

Figure S1. Factor V module membership identifies genes that correlate with severe COVID-19. Related to Figure 1 and Figure 2. Genes comprising the Factor V module are graphed. The x axis represents the correlation of a given gene's expression with the module eigenvalue. The higher the correlation, the more representative the module expression is of the gene. The Y axis represents the correlation between a gene's expression and disease status (HC versus severe COVID-19 at 0-12 days post symptom onset). The higher the correlation, the more able a given gene can distinguish severe COVID-19 from health. The red lines demarcate the "hub genes" which represent genes that strongly model the module and disease status. The genes illustrated are: KIF1B; PGD; PADI4; SORT1; PHTF1; SLC22A15; S100A9; F5; QSOX1; CHIT1; MTARC1; NLRC4; AGFG1; LTF; CAMP; GYG1; PLD1; ATP11B; ANXA3; CARD6; MCTP1; CYSTM1; SLC36A1; HK3; SERPINB1; SRPK1; MAPK14; VNN1; ABCA13; GRB10; SLC37A3; CLEC5A; BMX; ADAM9; ASPH; UGCG; LCN2; TCN1; MMP8; CSGALNACT2; CLEC4D; PLBD1; FGD4; IRAK3; DRAM1; CKAP4; CAMKK2; KLFV; ATP8B4; ITGAM; HP; HPR; WSB1; MPO; MAFG; RAB31; PSTPIP2; BPI; MMP9; RETN; MCEMP1; CEACAM8; CD177; PGLYRP1; FCAR; CLTCL1; RGL4; TSPO; SAMSN1; KCNE1; TRPM2.

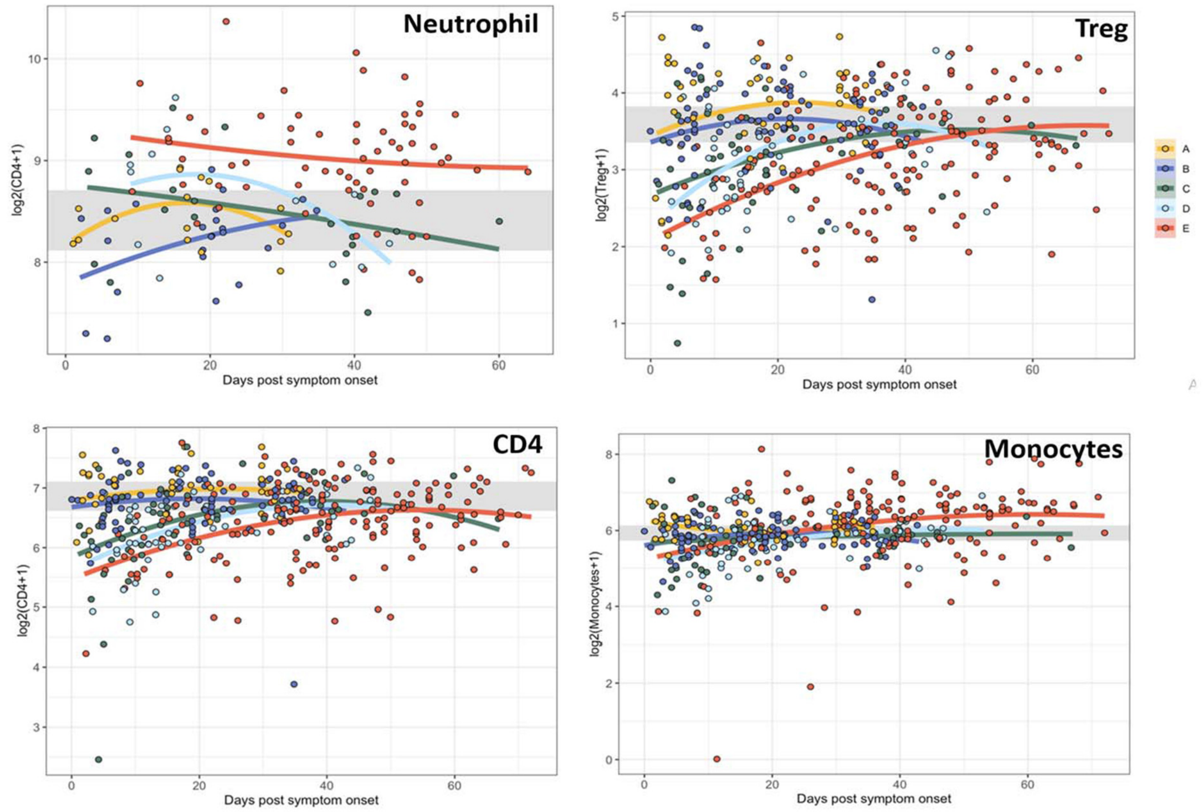

Figure S2. Leukocyte cell types have different trajectories over time. Related to Figure 2. Mixed-effect model with quadratic time trend showing the longitudinal trajectories for neutrophils, Tregs, CD4 and monocytes over time. A, HCW screening asymptomatic; B, HCW screening symptomatic; C, hospitalised mild disease; D, hospitalised requiring oxygen; E, hospitalised, intensive care.

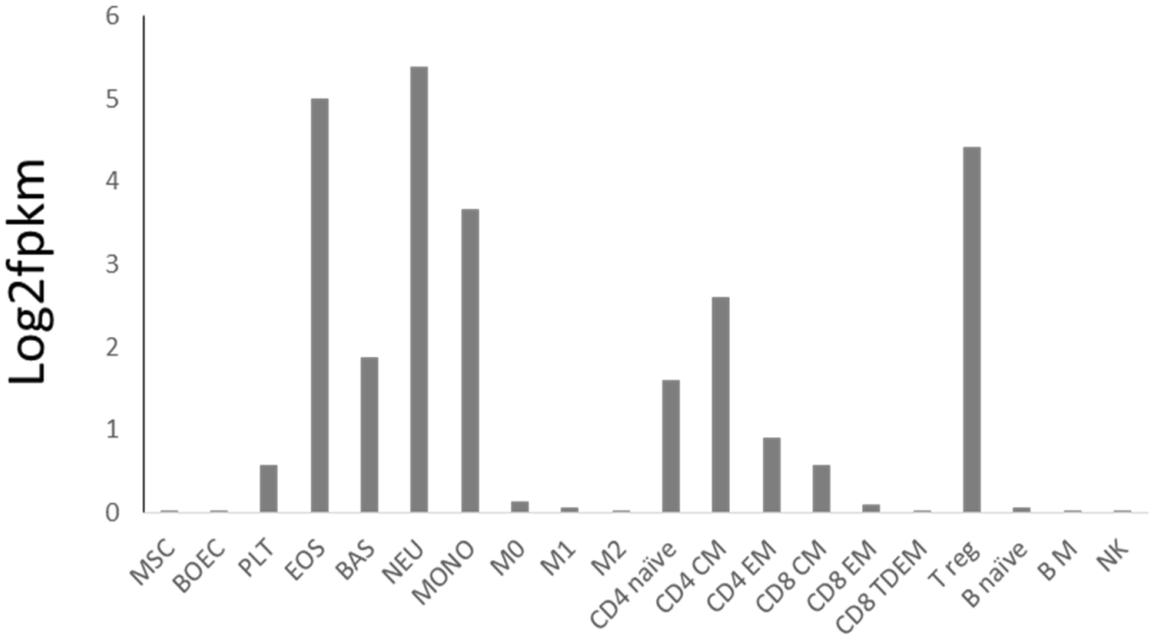

Figure S3. FV is expressed in peripheral blood leukocytes. Related to Figure 1.

Analysis of the Blueprint (https://www.blueprint-epigenome.eu/) database identified FV expression in peripheral blood cell subsets in healthy individuals, with the highest expression in neutrophils, eosinophils, monocytes and Tregs derived from healthy individuals. fpkm - fragments per kilobase of transcript per million mapped reads. MSC: mesenchymal stem cells, BOEC: blood outgrowth endothelial cells, PLT: platelet, EOS: eosinophil, BAS: basophil, NEU: neutrophil, MONO: monocyte, M0, M1 and M2: macrophages, CM: central memory, EM: effector memory, TDEM: terminally differentiated effector memory, T reg: regulatory T cells, B M: B memory

NK: natural killer.

Table S1. Gene Ontology (GO) enrichment analysis of FV module genes. Related to Figure 1.

Name	p-value	Adjusted p-value	Odds ratio	Combined score
Neutrophil mediated immunity (GO:0002446	6.500E-49	1.433E-45	13.97	1549.67
Neutrophil degranulation (GO:0043312)	3.299e-48	3.636e-45	13.92	1521.46
Neutrophil activation involved in immune response (GO:0002283)	5.879e-48	4.319e-45	13.78	1498.52
Innate immune response in mucosa (GO:0002227)	1.773e-8	0.000005583	32.91	587.37
Mucosal immune response (GO:0002385)	1.647e-7	0.00004537	21.93	342.56
Defense response to Gram-negative bacterium (GO:0050829)	1.061E-9	4.675e-7	13.14	271.47
